# [6*S*]-5-Methyltetrahydrofolic Acid and Folic Acid Pregnancy Diets Differentially Program Metabolic Phenotype and Hypothalamic Gene Expression of Wistar Rat Dams Post-Birth

**DOI:** 10.3390/nu13010048

**Published:** 2020-12-25

**Authors:** Emanuela Pannia, Rola Hammoud, Rebecca Simonian, Erland Arning, Paula Ashcraft, Brandi Wasek, Teodoro Bottiglieri, Zdenka Pausova, Ruslan Kubant, G. Harvey Anderson

**Affiliations:** 1 Department of Nutritional Sciences, Faculty of Medicine, University of Toronto, Toronto, ON M5S 1A8, Canada; e.pannia@mail.utoronto.ca (E.P.); rola.hammoud@mail.utoronto.ca (R.H.); rebecca.simonian@mail.utoronto.ca (R.S.); zdenka.pausova@utoronto.ca (Z.P.); 2 Center of Metabolomics, Institute of Metabolic Disease, Baylor Scott and White Health, Dallas, TX 75246, USA; erland.arning@bswhealth.org (E.A.); paula.ashcraft@bswhealth.org (P.A.); brandi.wasekpatterson@bswhealth.org (B.W.); teodoro.bottiglieri@bswhealth.org (T.B.); 3 Department of Physiology, Faculty of Medicine, University of Toronto, Toronto, ON M5S 1A8, Canada; 4 The Hospital for Sick Children, Toronto, ON M5G 1X8, Canada

**Keywords:** folate, folic acid, pregnancy, food intake regulation, hypothalamus, postpartum, weight-gain

## Abstract

[6*S*]-5-methyltetrahydrofolic acid (MTHF) is a proposed replacement for folic acid (FA) in diets and prenatal supplements. This study compared the effects of these two forms on maternal metabolism and hypothalamic gene expression. Pregnant Wistar rats received an AIN-93G diet with recommended FA (1X, 2 mg/kg, control), 5X-FA or equimolar levels of MTHF. During lactation they received the control diet and then a high fat diet for 19-weeks post-weaning. Body weight, adiposity, food intake, energy expenditure, plasma hormones, folate, and 1-carbon metabolites were measured. RNA-sequencing of the hypothalamus was conducted at parturition. Weight-loss from weaning to 1-week post-weaning was less in dams fed either form of the 5X vs. 1X folate diets, but final weight-gain was higher in 5X-MTHF vs. 5X-FA dams. Both doses of the MTHF diets led to 8% higher food intake and associated with lower plasma leptin at parturition, but higher leptin at 19-weeks and insulin resistance at 1-week post-weaning. RNA-sequencing revealed 279 differentially expressed genes in the hypothalamus in 5X-MTHF vs. 5X-FA dams. These findings indicate that MTHF and FA differ in their programing effects on maternal phenotype, and a potential adverse role of either form when given at the higher doses.

## 1. Introduction

The regulation of food intake, body weight, stress response, and circadian cycles is highly reliant on the development and plasticity of the hypothalamus to enable proper integration of central- and peripheral-derived signals and nutrient-related cues [1,2,3]. In addition to the fetus and newborn, the maternal brain undergoes dynamic functional adaptations throughout pregnancy into lactation [4]. As such, the peripartum period represents a period of high susceptibility to environmental factors that may influence mothers’ later-life outcomes. Folate is an essential B-vitamin required during pregnancy to support both maternal and fetal health outcomes [5] and regulatory functions of the brain [6,7]. Because of the biological importance of folate during pregnancy, deficiencies and/or excessive intakes may alter central or peripheral regulatory systems perpetuating the development of long-term health consequences [8,9,10].

To date, several studies demonstrated the programming potential of high FA towards negative metabolic health outcomes in the offspring. In the rodent model, exposure to FA at 2.5–10X above requirements during pregnancy resulted in male offspring that had higher body weight, food intake and central and/or peripheral metabolic dysregulation [11,12,13,14]. In contrast, female offspring born to high FA fed mothers exhibited lower post-weaning body weight-gain [15,16], but similarly showed evidence of metabolic dysregulation, including reduced bone length, mineral content and density that associates with their lower weight-gain [17]. While the effects of FA intake on maternal health outcomes have been investigated to a lesser extent, our recent study showed that 2.5–10X-FA intakes during pregnancy also lead to metabolic dysregulation in the mother early post-birth [18], and others have shown 20X-FA provided during pregnancy and lactation leads to the development of obesity in the postpartum mother and future mothers to be (i.e., female offspring) [19].

In North America, many women consume FA at 2.5–7-fold the tolerable upper intake level of 1000 µg/day [20,21], raising concern of the potential negative effects associated with high intakes. 5-methyltetrahydrofolate (5-MTHF) is the bioactive form of folate derived from the reduction of FA or 5-MTHF naturally present in foods. 5-MTHF participates in the biosynthesis of nucleotides and monoamine neurotransmitters, homocysteine (Hcy) metabolism and production of S-adenosylmethionine (SAM). SAM is the universal methyl donor involved in cellular methylation reactions of which regulates dynamic changes in gene expression [22]. However, the conversion of FA to 5-MTHF is limited by common polymorphisms as well as high FA-induced enzyme inhibitions [23]. High concentrations of serum [24] and maternal cord blood [25] unmetabolized FA have been detected and predicted to affect the metabolism, intracellular transport and/or regulatory functions of bioactive folates [26,27].

As a result, the replacement of FA with the reduced bioactive form of folate in dietary supplements has been proposed [9,23,28]. Many prescribed and over-the-counter prenatal supplements now incorporate the naturally occurring calcium salt, [6*S*]-5-methyltetrahydrofolic acid (MTHF), at a dose that is equivalent to those containing FA (1000 µg) [9,28]. Moreover, its addition to baby formula has been discussed [29]. Support for the use of MTHF is shown through randomized clinical trials that showed MTHF is equally or more effective as FA at increasing blood folate concentrations required for birth defect prevention [30]. Furthermore, MTHF supplementation has been shown to correct low 5-MTHF production arising from genetic polymorphisms in folate-metabolism genes or FA-induced enzyme inhibitions [23,31]. The therapeutic potential of MTHF compared to FA in the prevention and possible treatment of mental health issues including perinatal depression has also been reported [32], further encouraging the replacement of FA with MTHF during pregnancy. However, a comparison of the effects of the different folate forms provided during pregnancy and on long-term metabolic health has not been conducted.

The objective of this study was to compare the effects of diets containing FA at recommended (1X) or high (5X) doses vs. equimolar MTHF provided during pregnancy on the post-partum metabolic phenotype of Wistar rat mothers. In addition, we also aimed to investigate whether high folate diets differentially affected hypothalamic energy regulatory genes and pathways in the mother immediately following parturition. We hypothesized that the adverse long-term effects of high FA intakes on mothers are not observed with high MTHF additions to the pregnancy diet, and these effects associate with modified expression of regulatory genes in the hypothalamus. Primary outcome measure was body weight-gain. Other outcome measures included long-term food intake, energy expenditure, plasma energy regulatory hormones, plasma and tissue folates and 1-carbon metabolites and hypothalamic gene expression measured at parturition and up to 19 weeks post-weaning.

## 2. Materials and Methods

### 2.1. Animals and Diets

All animal work was approved by the University of Toronto Animal Care Committee (#20011892). First-time pregnant Wistar rats (2–3 days, 170–250 g) were purchased from Charles River Farms (Quebec, Canada) and individually housed upon arrival. Animals were maintained on a reverse 12-h dark-light cycle (lights on at 07:00 a.m. at 22 ± 1 °C) and *ad libitum* food and water were provided throughout the study. Dams were randomly allocated to one of four dietary interventions that were provided contemporaneously and only during pregnancy until term (3 weeks). Pregnant dams (*n* = 16–18/group) received an isocaloric AIN-93G diet (energy density 4.0 kcal/g) [33] containing either the recommended (1X, 2 mg/kg diet, control) or 5X-quantity of FA for rodents (1X-FA or 5X-FA) or equimolar recommended levels of [6*S*]-5-methyltetrahydrofolic acid, calcium salt (1X, 2.1 mg/kg diet, Metafolin^®^, Merck & Cie, Schaffhausen, Switzerland) or 5X-quantity of MTHF (1X-MTHF or 5X-MTHF). FA and MTHF diets at equimolar doses have been used in clinical trials assessing folate status and related-health effects [34,35,36]. The recommended (1X) dose was chosen based on the FA requirements for rodents for adequate growth rate [37] and reflects the FDA requirements for non-pregnant women (400 µg/day) [38]. The 5X dose was chosen to reflect FA intakes commonly consumed in North America above the basal dietary requirements for humans [20,21]. Diets were custom made by Research Diets Inc. (New Brunswick, NJ, USA) (Supplementary Materials Table S1) and stored at 4 °C and in the dark until provided fresh weekly. The additions of the MTHF form of folate into foods has been shown to be stable at both 4 °C and room temperature for up to 24 months [39]. Analytical testing of the high folate diets following a 36-month storage at −80 °C showed >80% retention of folates (Table S1), further confirming their long-term stability.

At birth, a subset of dams (*n* = 5–6/group) and their entire litters were terminated for analyses. Of the remaining dams, within 24 h post-birth, litters were culled to 6 pups/dam (3 male/female) for the lactation period (3 weeks). Dams were then all fed the control FA diet (1X-FA) during lactation until weaning (21 days post-birth). Dams (*n* = 10–12/group) were maintained on a high fat diet (45% kcal from lard), adjusted to provide a similar micronutrient content per 100 kcal as the control (1X-FA) AIN-93G diet, until termination at 19-weeks post-weaning. As post-weaning body weight-gain was our primary outcome measure, a sample size of *n* = 10–12/group was used to achieve statistical power for detecting 10% difference among treatment groups (α = 0.05, β = 0.8).

### 2.2. Body Weight, Food Intake, Body Composition and Plasma Analyses

Body weight of dams and pups were recorded immediately following parturition and body weight of dams were recorded thereafter at weaning and weekly until 19-weeks post-weaning. Food intake was recorded weekly. Early weight-loss of the mothers was calculated as the difference between 1-week post-weaning and weaning as previously reported [18], and maternal weight-gain calculated from 1–19 weeks post-weaning. The 1-week post-weaning time point was chosen as it is a hypothesized time point when maternal metabolism begins to re-stabilize to the pre-pregnancy set-point or establishment of a new set-point [40]. All terminations occurred by rapid decapitation following an 8–10 h daytime fast. The intra-abdominal (visceral) adipose tissue (VAT), which constituted retroperitoneal, perirenal and periovarian fat pads, were manually excised and weighed. Adiposity Index (AI%) was calculated as percentage of total VAT weight adjusted per gram of body weight. Liver weight was also calculated as a percentage per gram of body weight, and percentage of total liver lipids were determined according to the method described by Folch et al. [41] and expressed per gram of tissue weight. Whole brains were excised and immediately frozen on dry ice for later analyses. Trunk blood from dams at parturition and 19-weeks post-weaning were collected and plasma was separated and immediately frozen at −80 °C for further analyses.

### 2.3. Folate and Related 1-Carbon Metabolites

Concentrations of plasma and tissue folates [42,43] along with related 1-carbon metabolites including methionine, SAM, S-adenosylhomocysteine (SAH), cystathionine (Cys), choline and betaine [44] were measured in dams at parturition and at 19-weeks post-weaning by liquid chromatography tandem mass spectrometry (LC-MS/MS). Plasma Hcy was also measured by LC-MS/MS as previously described [45].

### 2.4. Plasma Hormones, and Insulin Tolerance Test

At parturition and 19-weeks post-weaning, fasting plasma glucose, insulin, active ghrelin and leptin were measured by commercially available assays: glucose (Cat#10009582, Cayman Chemical Co., Ann Arbor, MI, USA), insulin (Cat# 80-INSRT-E01, Alpco, Salem, NH, USA), leptin (Cat# EZRL-83K, EMD Millipore, Billerica, MA, USA) and active ghrelin (Cat#EZRGRA-90K, EMD Millipore, Billerica, MA, USA). Plasma leptin concentrations were further adjusted for VAT mass. Insulin resistance (IR) at parturition and 19-weeks post-weaning was evaluated via a surrogate index of the homeostatic model assessment insulin resistance (HOMA-IR) calculated as follows: [fasting glucose (in mg/dL) × fasting insulin (in µU/mL)]/2430] [46]. An insulin tolerance test (ITT) was also conducted in a subset of dams at 1- and 12-weeks post-weaning (*n* = 8/group) by measuring the glucose concentrations following a 6-h day-time fast and intraperitoneal injection of an insulin bolus (0.75 IU of insulin/kg body weight). Glucose was measured with the Accu-Chek^®^ Aviva glucometer (Roche Diagnostics, Laval, QC, Canada) at 0, 15, 30, 60, 90 and 120 min via tail prick. To better ascertain the function of insulin-stimulated glucose uptake by tissue and insulin-stimulated hepatic glucose suppression, the rate constant for glucose disappearance (kITT) was calculated 0–60 min using the formula kITT (%min^−1^) = (0.693/t^1/2^) × 100.

### 2.5. Energy Expenditure

Whole-body energy expenditure using the Comprehensive Lab Animal Monitoring System (CLAMS™, Columbus Instruments, Columbus, OH, USA) was measured in a subset of dams (*n* = 8/group) as previously reported [47]. Briefly, animals were transferred to The Hospital for Sick Children (Toronto, ON, Canada) at 14 weeks post-weaning, allowed a 1-week acclimatization period and tested on week 15. All measurements were taken at room temperature (22 °C) and each animal was monitored for two consecutive 24-h periods (24-h acclimatization, 24-h data collection and analysis), as described elsewhere [26,27]). Heat production was automatically calculated from O_2_ consumption (VO_2_) and CO_2_ production (VCO_2_) as follows: heat (kcal/kg/h) = (3.815 + 1.232 × (VCO_2_/VO_2_)) × VO_2_ (mL/kg/h) and animal activity was measured using the Opto-M3 activity monitor (Columbus Instruments, Columbus, OH, USA).

### 2.6. Brain Analyses from 5X-FA and 5X-MTHF Dams

#### 2.6.1. Brain Dissections and RNA-Sequencing

The primary nutrient-sensing nucleus of the hypothalamus, the arcuate nucleus (ARC), was identified and macrodissected from frozen whole brain tissue collected from 5X-FA and 5X-MTHF dams. The ARC was excised using the approximate stereotaxic coordinates according to “The Rat Brain in Stereotaxic Coordinates” (Bregma −2.12 mm to −2.56 mm) [21], as previously described [40]. The optic chiasm was used as a landmark for the anterior portion of the hypothalamus and as a horizontal limit for the first cut that was made using a sterile single edge razor blade. The ARC was isolated by inserting a blade ~2 mm caudal to the first slice. The collected coronal brain slice was placed on a glass slide over dry ice and dissociated free hand. The 3rd ventricle was used as an anatomic reference guiding the excision of the ARC for RNA extraction. The same coronal sections were also used for collection of a representative region of the midbrain for processing of folate [42] and 1-carbon metabolites [38] by LC-MS/MS, as previously described. This brain section was excised by creating a box cut on either side of the midline that bordered the dorsal third ventricle and contained regions of the mediodorsal thalamic nucleus.

For RNA analyses, the dissected ARC at parturition (*n* = 5/group) was homogenized on ice using a tissue rupture homogenizer (Qiagen Tech., Mississauga, ON, Canada). RNA was isolated using Trizol reagent and chloroform extraction according to the manufacturer’s protocol (Invitrogen, Grand Island, NY, USA) and quantified by NanoDropTM2000 Spectrophotometer (Thermo Scientific Inc., Wilmington, DE, USA). Quality of RNA was assessed using the Agilent BioAnalyzer (RNA 6000 Nano LabChip) at the Centre for Applied Genomics (TCAG) at the Hospital for Sick Children. Whole-transcriptome analysis by RNA-sequencing was performed at TCAG at the Hospital for Sick Children using a poly-A selection of mRNA. Paired-end 2 × 125 bp sequencing was performed with Illumina HiSeq 2500 instruments at a sequencing depth of ≈20 million paired reads per sample. Sequence data was demultiplexed and base calls were converted to FASTQ format with bcl2fastq2 v2.20. The sequence read quality was assessed using FastQC v.0.11.5 [48]. Adapter trimming and removal of lower quality ends was performed using Trim Galore v. 0.5.0 [49]. Trimmed reads were screened for presence of rRNA and mtRNA sequences using FastQ-Screen v.0.10.0 [50]. RSeQC package v.2.6.2 [51] was used to confirm strand specific RNA-Seq library construction and to assess positional read duplication and read distribution across exonic, intronic and intergenic regions. The quality of trimmed reads was re-assessed with FastQC. STAR aligner v.2.6.0c [52,53] was used to align the trimmed reads to Rnor_6.0 genome downloaded from Ensembl database version 98.6 using Ensembl gene models. The STAR alignments were processed to extract raw read counts for genes using htseq-count v.0.6.1p2 (HTSeq) [54]. Two-condition differential gene expression was performed with DESeq2 [55] v. 1.26.0s, using R v.3.6.1. Initial minimal filtering of 10 reads per gene for all samples was applied to the datasets. More strict filtering to increase power is automatically applied via independent filtering on the mean of normalized counts within the DESeq results function. Selected differentially expressed genes (DEGs), corrected for multiple testing (Benjamini-Hochberg), were used as an input for pathway analysis (*p*-adjusted, *padj* ≤ 0.05). The reference level was data from 5X-FA dams.

#### 2.6.2. Enrichment Analysis of Differential Expressed Genes (DEGs)

Goseq procedure was used for pathway enrichment analyses. For each goseq analysis, two lists of genes were created with up-regulated genes (log fold change > 0) and down-regulated genes (log fold change < 0) using *padj* ≤ 0.05. The up- or down-regulated gene lists were used one at a time with correction for gene length bias. Rat gene sets were obtained from GO (through R package GO.db, 3.8.2); KEGG files were downloaded directly from the KEGG website on 2020/03/26; Reactome sets were downloaded from the Reactome website, and used together with GO and KEGG sets to create a custom gene set collection; all sets were filtered to retain those with several genes between 5 and 1000. To use the custom gene set collection, the gene2cat argument was set to point to the gene set collection, and used together with the output of nullp in the goseq function to estimate the probability of over and under-representation in each set, for either up- or down-regulated lists of genes using Wallenius approximation to estimate the null distribution. Genes in the differential analysis that could not be mapped to any gene set term were excluded from the background. The enrichment results for gene sets enriched in up-regulated and down-regulated genes or both (up- and down-regulated) were generated using *p* < 0.005 and *padj* < 0.1. Results were visualized in cytoscape using the enrichment map plugin [56]. The cytoscape map was analyzed using the autoannotate plug in with default parameters. Cluster names were manually adjusted to better reflect the underlying gene sets. This data set is available at the NCBI Gene Expression Omnibus (GEO) under accession GSE161954.

#### 2.6.3. Validation of DEGs: cDNA Synthesis and qRT-PCR

Select genes in the ARC at parturition that were enriched in pathway gene sets related to energy metabolism were validated by qRT-PCR (*n* = 5/group) and also at post-weaning (*n* = 6–7/group). cDNA was synthesized using 1500 ng of total RNA using the High Capacity cDNA Archive Kit (Applied Biosystems Inc; ABI, Foster City, CA, USA) on the TProfessional Standard Gradient 96 thermocycler (Biometra). qRT-PCR was performed on the ABI PRISM 7900 Sequence Detection System using SYBR Green (Thermo Fisher). mRNA levels were normalized to levels of beta-2-microglobulin used as a housekeeping gene selected based on lowest variance in Cq value. Primers were designed using Primer-BLAST software and are listed in Table S2. mRNA fold change were analyzed using the 2^−∆∆CT^ method [57].

### 2.7. Statistical Analyses

Data were analyzed using SAS Version 9.4 software (SAS Institute Inc., Carey, NC, USA). A two-way analysis of variance (ANOVA) was performed using PROC GLIMMIX procedure with folate dose (1X vs. 5X) and form (FA vs. MTHF) as main factors and Dose × Form interaction term for measures of cumulative food intake, total body weight-gain, plasma hormones, insulin tolerance, folate, and 1-carbon metabolites. A covariate argument for “batch effect” was used for analyses of body weight and food intake to account for potential differences in stress response in dams who were transferred between institutions for energy expenditure analysis. To identify the effect of treatment during pregnancy on weight-change over time, a Repeated Measures ANOVA was used with pregnancy diet and time as the main factors and a Diet × Time interaction term. Comparisons made between brain gene expression and folate concentrations of only 5X-FA vs. 5X-MTHF dams were conducted using a Student’s *t*-test. All significant interactions were examined by Tukey’s post-hoc analysis. Statistical significance was declared at *p* < 0.05 and all data is expressed as least square (LS) Mean ± S.E.M.

## 3. Results

### 3.1. Effects of Folate Diets on Maternal Plasma and Liver 5-MTHF and 1-Carbon Metabolites at Parturition and 19-Weeks

Upon parturition, plasma FA was only detectable in dams fed 5X-FA diet (9.72 ± 1.21); all other dams (1X-FA, 1X-MTHF or 5X-MTHF) had FA concentrations below the lower detection limit of quantification. Plasma (Table 1) and liver (Table 2) 5-MTHF and methionine were higher in dams on 5X vs. 1X diets of either form (Dose *p* < 0.01). At 19-weeks post-weaning, plasma FA was below the detection limit in all groups, and plasma and liver 5-MTHF did not differ among the groups.

Plasma (Table 1) and liver (Table 2) concentrations of related 1-carbon metabolites were also affected by FA and MTHF diets at parturition. Plasma choline was higher in dams fed 1X vs. 5X folate diets of either form (Dose *p* < 0.05). Plasma betaine was higher in dams fed 5X-FA vs. 5X-MTHF diets, but liver betaine did not differ between the four groups. Plasma concentration of Hcy was higher in MTHF compared to FA dams at parturition (Form *p* < 0.05), along with a similar trend for its derivative Cys (*p* = 0.06). Accordingly, hepatic concentrations of SAH were higher in MTHF compared to FA dams (Form *p* < 0.05). By 19-weeks post-weaning, an interaction effect of folate form and dose was found on hepatic concentrations of SAM and SAH, but not other metabolites. While SAM was higher in 5X-FA compared to 5X-MTHF dams (*p* < 0.05), no differences in SAH were revealed once adjusted for multiple comparisons.

**Figure 1:**
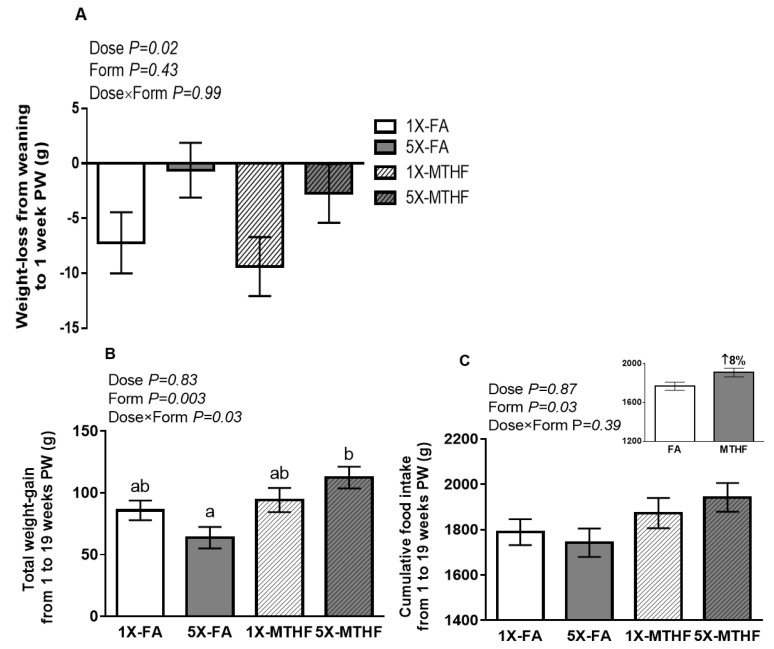
(**A**) Weight-loss from weaning to 1-week post-weaning (PW), (**B**) total weight-gain at 19-weeks PW and (**C**) cumulative food intake calculated from 1- to 19-weeks post-weaning (PW). *n* = 10–12/group. Values are Least Square Mean ± S.E.M. Analyzed by Two-way ANOVA. ^ab^ Significantly different by Tukey Post-hoc Analysis. Abbreviations: AIN-93G rodent diet with 1X (recommended, 2 mg/kg) or 5X folic acid (FA) or equimolar [6*S*]-5-methyltetrahydrofolic acid calcium salt (MTHF) at 1X (2.1 mg/kg) or 5X (10.4 mg/kg) levels.

Body weight of dams upon arrival and at parturition and up to 1-week post-weaning were not affected by folate dose or form consumed during pregnancy (Table S3). Cumulative food intake during pregnancy and lactation was also not different between diet groups. However, dams fed the 1X folate diets had greater weight-loss (Dose *p* < 0.05), correcting for pregnancy weight-gain, compared to 5X folate fed dams (Figure 1A).

The gestational diets did not affect litter size (1X-FA 12.9 ± 0.6, 5X-FA 12.5 ± 0.6, 1X-MTHF 12.5 ± 0.6, 5X-MTHF 11.8 ± 0.6) or the average birth weight of male (1X-FA 7.0 ± 0.2 g, 5X-FA 7.0 ± 0.2 g, 1X-MTHF 7.2 ± 0.1 g, 5X-MTHF 7.4 ± 0.2 g) or female (1X-FA 6.8 ± 0.1 g, 5X-FA 6.8 ± 0.1 g, 1X-MTHF 6.7 ± 0.2 g, 5X-MTHF 6.9 ± 0.2 g) pups; confirming no confounding effects of these variables on maternal later-life outcome.

While maternal body weight-gain from 1 to 19-weeks post-weaning was affected by folate form, the magnitude of change was dependent on the dose, as evidenced by a significant interaction effect (Dose×Form *p* < 0.05). Overall, dams fed MTHF diets during pregnancy gained ~30% more weight than FA dams; however only 5X-MTHF dams gained significantly more weight than 5X-FA dams (Figure 1B). Further investigation into the effect of the pregnancy diets over time revealed that weight-gain was significantly lower in 5X-FA dams compared to 5X-MTHF dams starting from week 15 up to week 19 post-weaning (Figure S1). While liver weight of 1X-MTHF dams was higher compared to 5X-MTHF at parturition (Dose×Form, *p* < 0.05, Table 3), by post-weaning week 19, adiposity, liver weight nor total liver lipids were affected between diet groups (Table 3). In contrast to the effects of folate diets on body weight, cumulative food intake from 1 to 19 weeks post-weaning was affected by folate form (Form *p* < 0.05) but not dose nor an interaction effect. Dams fed the MTHF pregnancy diets compared to FA consumed ~8% more high fat diet throughout the post-weaning period (Figure 1C).

### 3.2. Effects of Folate Diets on Energy Expenditure and Locomotor Activity

**Figure 2:**
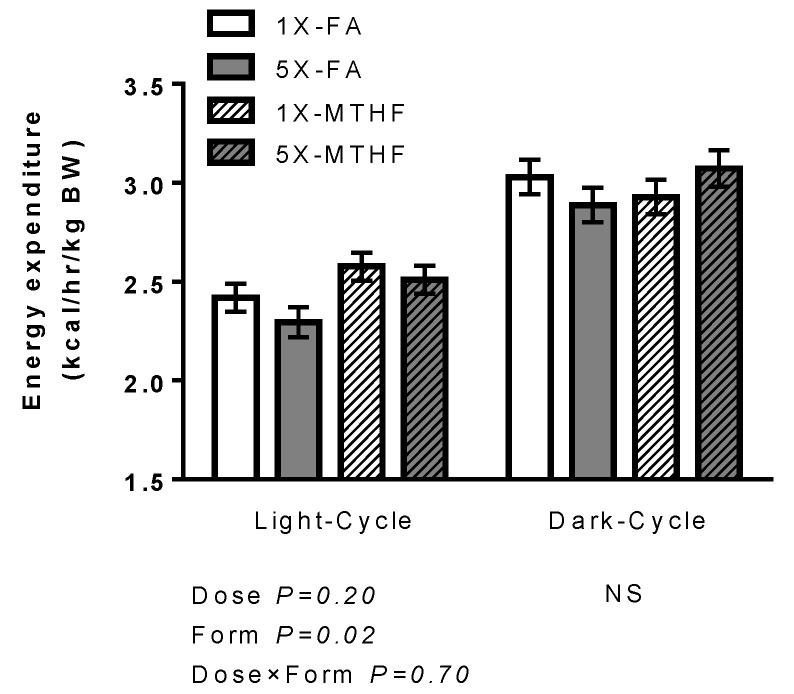
Average energy expenditure (kcal/kg/h) during 24 h in dams at 15-weeks post-weaning (PW). Embedded graph represents the effect of Form alone (FA vs. MTHF). *n* = 10–12/group. Values are Least Square Mean ± S.E.M. Analyzed by Two-way ANOVA, significance at *p* < 0.05. Abbreviations: AIN-93G rodent diet with 1X (recommended, 2 mg/kg) or 5X folic acid (FA) or equimolar [6*S*]-5-methyltetrahydrofolic acid calcium salt (MTHF) at 1X (2.1 mg/kg) or 5X (10.4 mg/kg) levels.

At 15-weeks post-weaning, MTHF dams had higher energy expenditure (kcal/h/kg BW) during the light cycle compared to FA dams (Form *p* < 0.05), but these differences were not maintained throughout the dark-cycle (Figure 2). Spontaneous activity (Figure S2) was not affected during the light cycle but there was an effect of activity during the dark-cycle attributable to folate dose that was dependent on the form, as evidenced by a significant Dose×Form interaction (*p* < 0.05). However, the magnitude of these changes was not sufficient to induce overall significant differences in energy expenditure during this time. While, 1X folate dams had lower stereotypy activity than 5X folate dams during the dark-cycle (Figure S2A), only 1X-MTHF dams had lower total activity (Figure S2B) attributable to a lower ambulation activity (Figure S2C) than 5X-MTHF dams. Respiratory exchange ratio, which reflects substrate oxidation, was not different between the groups (data not shown).

### 3.3. Effects of Folate Diets on Plasma Hormones and IR

Upon parturition, MTHF dams had lower plasma leptin compared to FA dams (Dose *p* < 0.001, Table 4). Plasma glucose and insulin concentrations and IR, as assessed by HOMA-IR, were not different between dams at parturition. At 1-week post-weaning, IR assessed by the rate of glucose disappearance (kITT), was lower in MTHF dams compared to FA dams (1X-FA 1.07 ± 0.13, 5X-FA 0.97 ± 0.12, 1X-MTHF 0.66 ± 0.12, 5X-MTHF 0.83 ± 0.12, (Form *p* < 0.05, Figure S3A,B); however no differences were observed at 12-weeks post-weaning (1X-FA 0.9 ± 0.09, 5X-FA 0.8 ± 0.09, 1X-MTHF 0.8 ± 0.9, 5X-MTHF 0.7 ± 0.1). At 19-weeks post-weaning, plasma leptin was higher and active ghrelin was lower in MTHF compared to FA dams (Form *p* < 0.05, Table 4), but plasma insulin, glucose and HOMA-IR were not different.

### 3.4. Effect of 5X-FA vs. 5X-MTHF Diets on Whole-Transcriptome Gene Expression in the ARC at Parturition

To investigate the effects of high FA vs. MTHF gestational diets on the maternal brain, gene expression in the hypothalamic ARC was compared between 5X-FA and 5X-MTHF dams at parturition. The 5X dose was preferentially chosen as this dose reflects current high intakes of FA in the North American population [20,21]. Like FA, MTHF supplementation is becoming widespread, thus it is possible that intakes may also increase over time.

**Figure 3:**
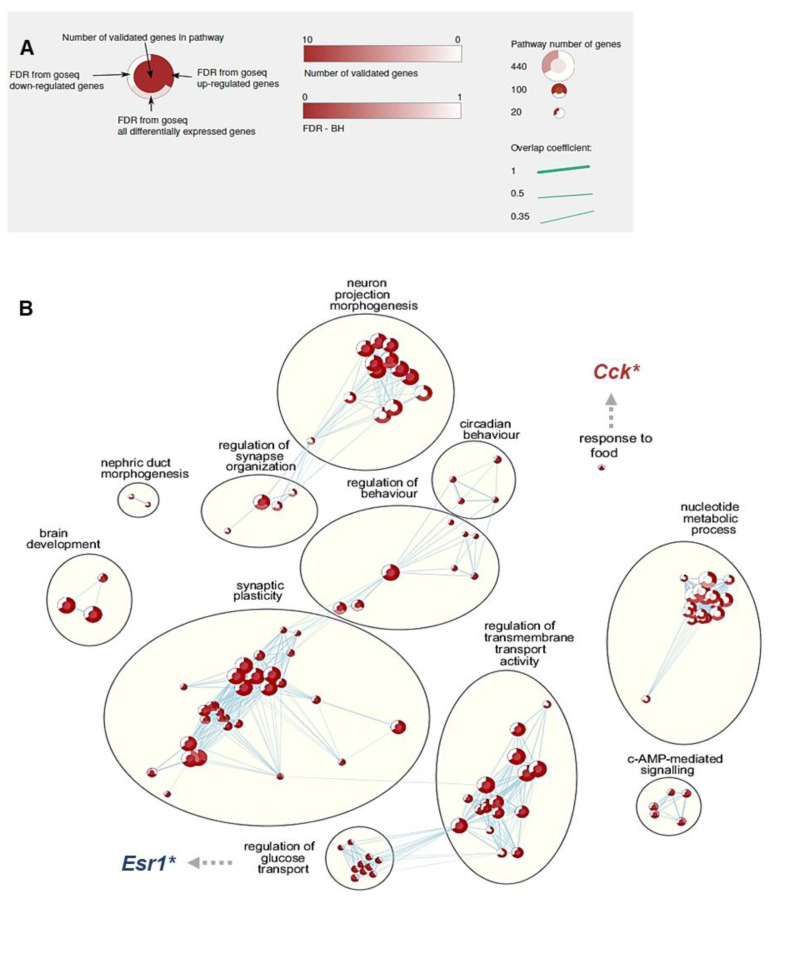

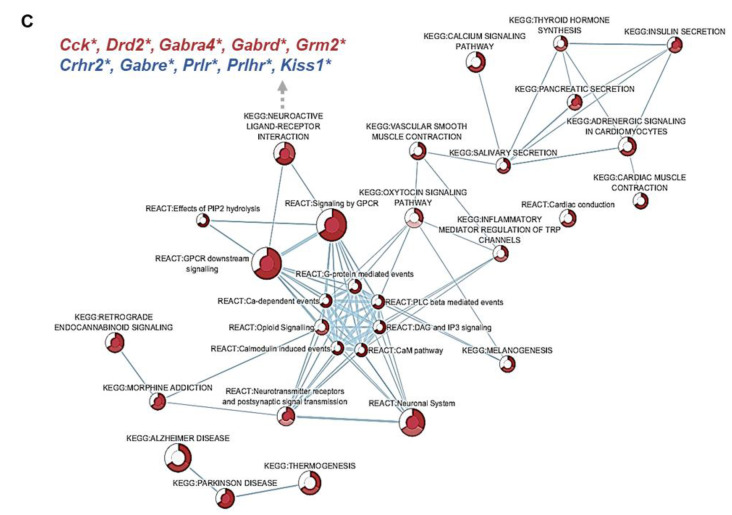
(**A**) Gene set enrichment legend, (**B**) Gene ontology (GO) and (**C**) Kyoto Encyclopedia of Genes and Genomes (KEGG) and Reactome pathways from custom gene set collections. Legend shows enrichment tests were run for gene sets enriched in “up-regulated”, “down-regulated” or “up- and down-regulated (i.e., differentially expressed)” genes in 5X-MTHF compared to 5X-FA dams using Goseq. Node size is proportional to the number of genes in the pathway set, and the connecting line thickness is proportional to the number of identical genes in connected sets. Outer node colors represent the FDR with multiple test correction (BH, Benjamini-Hochberg’s procedure). An FDR ≤ 0.1 and *p* ≤ 0.005 was used for enrichment analyses. Genes of interests (*) were validated by qRT-PCR at birth and post-weaning. Red font indicates if the gene was up-regulated and blue font indicates down-regulated genes. The center node color represents the number of identical genes that were from this validated gene list by qRT-PCR that are enriched in other pathways, with 10 being the maximum number of validated genes in a single pathway. Gene set clusters from GO are shown with representative cluster terms. Validated Gene Abbreviations: *Cck*, cholecystokinin; *Crh2*, corticotropin-releasing hormone receptor 2; *Esr1*, estrogen receptor 1; *Gaba4a*, gamma-aminobutyric acid type A (Gaba) receptor alpha; *Gabrd*, gaba receptor delta; *Gabre*, gaba receptor epsilon; *Grm2*, glutamate receptor 2; *Kiss1*, kisspeptin; *Prhr*, prolactin hormone receptor; *Prlhr*, prolactin-releasing peptide.

Upon parturition, a total of 279 differentially expressed genes were identified in the ARC of dams fed 5X-MTHF diets vs. 5X-FA diets during pregnancy. These genes included 170 up-regulated and 109 down-regulated genes (Table S4). The up-regulated and/or down-regulated genes were then classified for enrichment by GO, KEGG and Reactome pathway analyses. Gene sets in GO analyses identified 116 in Biological Processes, 24 in Molecular Function and 27 in Cellular Component (Tables S5 and S6). In Biological Processes (Figure 3B), up-regulated gene sets were mainly those related to ‘regulation of behaviour’, ‘circadian behaviour’ and ‘nucleotide metabolic processes’, while down-regulated gene sets were enriched only in ‘regulation of glucose transport’. Analysis of gene sets enriched in both up- and down-regulated genes included those related to ‘response to food’, ‘synaptic plasticity’ and ‘regulation of transmembrane transport’. In the Cellular Component, the enriched terms were also related to neurons and synapses and included ‘postsynapse’, ‘neuronal cell body’, ‘dendrite’ and ‘synaptic membrane’ that were enriched primarily due to up-regulated gene sets. Up- and down-regulated gene sets clustered around ‘GABAergic synapse’. In the Molecular Function cluster, up-regulated gene sets were enriched in several terms related to transporter activity and down-regulated gene sets were enriched in pathways related to hormone binding. Up- and down-regulated gene sets in Molecular Function were significantly enriched in ‘receptor ligand activity’.

A total of 30 gene sets were further enriched in KEGG and Reactome pathways (Figure 3C, Table S7) with 16 gene sets enriched in up-regulated genes related to neurological disorders and endocrine control and included ‘Parkinson’s disease’, ‘Alzheimer’s disease’, ‘opioid signaling pathway’, ‘calcium signaling pathway’, ‘thermogenesis’, ‘melanogenesis’, ‘insulin secretion’, ‘retrograde endocannabinoid signaling’ and ‘neuronal systems’. Gene sets enriched in both up- and down-regulated genes included gene sets related to neurotransmission, including ‘neuroactive ligand-receptor interaction’ pathway.

### 3.5. Validation of RNAseq Data Using qRT-PCR

**Figure 4:**
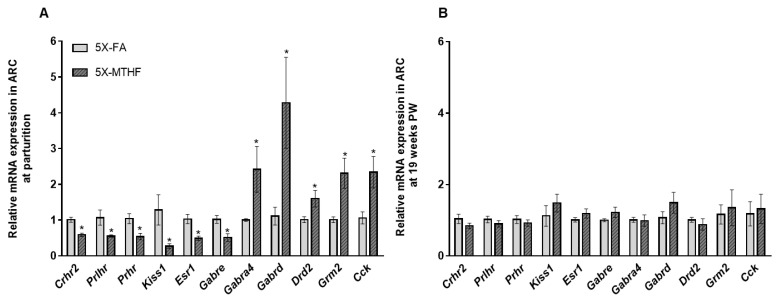
(**A**) Relative mRNA expression of differentially expressed genes identified by RNAseq validated by qRT-PCR in the ARC of 5X-MTHF compared to 5X-FA dams at parturition and (**B**) at 19-weeks post-weaning (PW). *n* = 5/group at parturition and *n* = 7/group post-weaning. Values are Means ± S.E.M. Comparisons between 5X-MTHF and 5X-FA groups were analyzed by Student T-test, significance at * *p* < 0.05. Abbreviations: AIN-93G rodent diet with 5X folic acid (FA) or equimolar [6*S*]-5-methyltetrahydrofolic acid calcium salt (MTHF) at 5X (10.4 mg/kg) levels. *Cck*, *cholecystokinin*; *Crh2*, *corticotropin-releasing hormone receptor 2*; *Esr1*, *estrogen receptor 1*; *Gaba4a*, *gamma-aminobutyric acid type A* (*Gaba*) *receptor alpha*; *Gabrd*, *gaba receptor delta*; *Gabre*, *gaba receptor epsilon*; *Grm2*, *glutamate receptor 2*; *Kiss1*, *kisspeptin*; *Prhr*, *prolactin hormone receptor*; *Prlhr*, *prolactin-releasing peptide*.

A total of 11 selected differentially expressed genes were validated with qRT-PCR. These genes were selected as they were found to be enriched in several key pathways regulating physiological changes during pregnancy and/or food intake regulation (Tables S6 and S7 ). These genes include *corticotropin-releasing hormone 2* (*Crh2*), *prolactin-releasing peptide receptor* (*Prlhr*), *prolactin hormone receptor* (*Prlr*), *Kisspeptin* (*Kiss1*), *Estrogen receptor 1* (*Esr1*), *Cholecystokinin* (*Cck*), *Gamma-Aminobutyric Acid Type A Receptor Subunit*- *alpha4* (*Gabra4*), -*delta* (*Gabrd*) and *-epsilon* (*Gabre*), *Dopamine receptor 2* (*Drd2*), and *Glutamate metabotropic receptor 2* (*Grm2*). RNAseq results showed a strong and positive correlation with genes validated by qRT-PCR (Figure S4A,B) confirming high reliability of the RNA-seq data set. While mRNA levels of *Crh2*, *Prlhr*, *Prhr*, *Kiss1*, *Esr1*, and *Gabre* were significantly down-regulated in ARC of MTHF dams compared to FA dams at parturition, mRNA levels of *Gabra4*, *Gabrd*, *Drd2*, *Grm2 and Cck* were significantly up-regulated in MTHF dams (Figure 4A, *p* < 0.05) corroborating RNAseq results. By 19-weeks post-weaning however, gene expression of the selected target genes in the ARC of dams were not affected (Figure 4B).

### 3.6. Effect of 5X-FA *vs.* 5X-MTHF Diets on Brain 5-MTHF and 1-Carbon Metabolites in Dams at Parturition and 19-Weeks Post-Weaning

Maternal brain concentrations of 5-MTHF (Table 5) were not different between diet groups at parturition or at 19-weeks post-weaning. While brain betaine concentrations were ~53% higher in 5X-FA compared to 5X-MTHF dams (*p* < 0.05), no other metabolites were affected.

## 4. Discussion

These results are the first to show that FA and MTHF when fed during pregnancy differ in their effects on programming of post-birth maternal metabolic phenotype and hypothalamic regulatory genes. Contrary to our hypothesis, the results provide evidence of a potential adverse role of both forms when fed at the higher dose. Firstly, post-birth weight-loss from weaning to 1-week post-weaning in dams was delayed. Although long-term body weight-gain was higher in 5X-MTHF compared to 5X-FA dams fed the high fat diet for 19-weeks, the increased weight did not reflect in a negative body composition phenotype. Second, MTHF diets led to higher PW food intake and associated with lower plasma leptin at parturition and higher leptin at 19-weeks post-weaning as well as IR at 1-week post-weaning. In addition, RNAseq analysis revealed 279 newly identified DEGs in the ARC of mothers at parturition in response to the high 5X-MTHF compared to 5X-FA pregnancy diet that were associated with dysregulated 1-carbon metabolism at parturition and later-life phenotypes.

The folate dose and form in the gestational diets resulted in different effects on body weight changes in the dams. Weight-loss from weaning to 1-week post-weaning was less in dams fed either form of the 5X compared to 1X folate diets. These results are consistent with our previous findings that a high FA dose during pregnancy may lengthen the period of metabolic adjustment that follows immediately post-birth [18] and report a novel observation that these effects are independent of folate form. However, after 19-weeks of the high fat diet, those on the high (5X) FA diet gained ~75% less weight than 5X-MTHF dams. Surprisingly, differences in body weight were not reflected in body composition, as they were independent of changes in visceral adiposity, liver weight or total liver lipids at 19-weeks.

In addition to higher body weight-gain up to 19-weeks post-weaning, the MTHF folate diets resulted in 8% higher food intake and associated with a metabolic profile early post-birth that favors increased food intake. Upon giving birth, MTHF mothers had lower plasma leptin, which may act as a long-term afferent signal in the negative-feedback loop of the hypothalamus to stimulate feeding [58]. At 1-week post-weaning, MTHF dams also had a lower constant for the disappearance of glucose (kITT), indicating transiently higher IR once exposed to the high fat diet. By 19-weeks post-weaning, plasma leptin normalized for VAT was higher in MTHF dams suggesting possible development of leptin resistance [59]. Moreover, MTHF dams had lower plasma levels of the orexigenic hormone ghrelin, which may be compensatory to their increased food intake and body weight as observed in overweight individuals [60] and high fat diet-induced obesity models [61].

As further evidence of effects of folate form on energy regulation, MTHF dams had higher energy expenditure during the light cycle, a period when rodents maintained on a high fat diet are known to have higher thermogenesis. This has been suggested to be mediated by an increase in leptin and its actions on the sympathetic nervous system to decrease feeding-bouts during this time [62,63]. These results point to a role of folate in affecting non-exercise activity thermogenesis, as measures of spontaneous activity were similar between diet groups during the light cycle and plasma leptin was accordingly higher in MTHF dams post-weaning. Together, these findings suggest that higher energy expenditure in MTHF dams may have been a homeostatic mechanism to counteract effects of increased food intake on their weight-gain.

To begin to investigate the association between the phenotypic effects of excess intakes of folates with the maternal brain transcriptome, RNAseq was conducted in the ARC of high (5X) folate fed dams immediately after giving birth. Our results showed that the 5X-MTHF pregnancy diet resulted in differential expression of 279 genes in the ARC of dams at parturition and enrichment of 197 regulatory pathways. While functional enrichment analysis revealed pathways that were diverse in function, many were related to regulation of behavior, food response, addiction, transmembrane signaling, synaptic plasticity, thermogenesis, and neuronal function, of which are relevant to the later-life observed postpartum phenotype of the dams in this study. Moreover, while the majority of the terms identified were enriched due to up-regulated gene sets, several regulatory pathways were also enriched due to both up- and down-regulated gene sets equally contributing to their enrichment (Table S4). These findings suggest that while 5X-MTHF supplementation during pregnancy may induce overall “activation” of hypothalamic gene expression compared to 5X-FA mothers at parturition, there also exists “dysregulation” in several central regulatory pathways controlling energy homeostasis.

Several affected hypothalamic regulatory pathways in 5X-MTHF compared to 5X-FA dams have been previously shown to be associated with obesity, mood disorders and addictive behavior, including food addiction [64,65]. These pathways include, but are not limited to, the KEGG neuroactive ligand-receptor interaction, GABAergic synapse, and retrograde endocannabinoid synapse. The neuroactive ligand-receptor interaction pathway is a group of receptors and ligands that regulate intracellular and extracellular signaling on the plasma membrane and is of particular interest as genes within this pathway are highly enriched (both activated and repressed) throughout several brain regions during pregnancy, parturition and the post-partum period in the rodent [66]. Many of the genes within this pathway also have a specific role in modulating effects of lactogenic hormones in preparation for lactation [67,68,69]. We identified candidate genes within the neuroactive ligand receptor interaction pathway to be validated as possible novel targets of folate-induced effects in the maternal brain during pregnancy, including *Crhr2*, *Prlhr*, *Kiss1*, *Gabra4*, *Gabre*, *Gabrd*, *Drd2* and *Grm2*. In addition, we also validated genes that were enriched in other related pathways that may play a role in mediating the observed maternal phenotype including *Esr1 and Cck*. While the selected genes have a role beyond food intake regulation, it is noteworthy that 5X-MTHF dams had transiently lower expression of genes known to be involved in the ARC to reduce food intake including *Crhr2* [70], *Prlhr* [71], *Kiss1* [72], and *Esr1* [73] and higher expression of genes encoding the GABA_A_ receptor involved in food stimulation [74] at parturition; possibly contributing to higher food intake over time in 5X-MTHF dams, especially once exposed to a highly palatable high fat diet during the post-weaning period. Moreover, our findings also highlight the interplay between folate-induced peripheral metabolic changes and molecular mechanisms in the brain as several of the identified genes have also been shown to be under the regulation of the plasma hormone leptin to regulate food intake (e.g., *Prlh* [75], *Drd2* [76], *Cck* [77]), the lactogenic hormone prolactin (e.g., *Prlr*, *Drd2* [69]) or modulate the fluctuating response of the ARC to circulating sex-steroids (e.g., *Gabre*, *Gabrd* [78]).

To offer additional insight towards the role of folate form during pregnancy in disease predisposition, we searched for common genes between our differentially expressed gene set and those identified in the disease center human database, Human Genome Epidemiology (HuGE) Navigator [79]. Disease terms related to the long-term phenotype of MTHF vs. FA dams and gene ontology terms identified by enrichment analysis were searched and gene lists were extracted and compared with our gene set (Table S8). Corroborating our phenotypic results, we identified 52 common genes between datasets using the search term *obesity*, 8 common genes related to *hyperphagia*, 10 genes related to *anorexia*, 59 genes related to *diabetes mellitus type-2* and 12 common genes related to *gestational diabetes*. Although this data cannot be interpreted as cause and effect, it is well known that the development of several metabolic and behavioral disorders is complex and commonly attributed to the dysregulation of genes and signaling pathways arising from multiple interacting regulatory systems. The differential expression gene set in this study may lead to hypothesis testing and the development of studies to identify novel candidate genes and pathways targeted by folate during pregnancy that have a role in disease on-set and/or prevention.

While the primary mechanism(s) by which folate dose and form during pregnancy affected maternal post-birth outcome are left uncertain, a probable mechanism is suggested by transient disruptions in folate-mediated 1-carbon metabolism upon giving birth that were re-stabilized by 19-weeks post-weaning. As expected, only the 5X-FA diet led to detectable amounts of plasma unmetabolized FA in the mother post-birth. As high unmetabolized FA have been linked to negative health consequences [27], this may have been a factor underlying differences in later-life phenotype that warrants investigation. Moreover, although plasma and hepatic concentrations of 5-MTHF and methionine at parturition directly reflected the dose of folate provided during pregnancy, with 5X dams with higher concentrations compared to 1X dams, potential perturbations in folate-mediated 1-carbon metabolism in plasma and tissues were also found that may explain differential programming of folate forms, especially at the higher dose.

Plasma and tissue concentrations of choline, betaine, SAH and Hcy at parturition were affected by the dose and form of folate provided during pregnancy; potentially contributing to the post-birth phenotype of the dams. Dams fed the MTHF diet had 1-fold higher plasma Hcy concentrations, which contrasts with clinical studies showing MTHF to result in lower plasma Hcy levels than FA in non-pregnant women [35,80]. However, the difference in Hcy due to folate form as found immediately post-birth in rat dams are likely specific to the effect of pregnancy on mediating transient changes in 1-carbon metabolism following parturition as found in mice [81]. Upon giving birth, maternal concentrations of choline and betaine are lower in the liver and increased in plasma to support placental transport to the fetus and milk production for lactation [82]. In turn, hepatic SAH hydrolase reaction is reduced, and endogenous choline synthesis is increased, both of which are suggested to be responsible for the transient increases in maternal plasma Hcy observed at the time of delivery. Accordingly, MTHF compared to FA dams also had higher hepatic concentrations of SAH that associated with higher plasma Hcy and a strong trend towards its derivative Cys (*p* = 0.06).

Because the 5X folate diets differed in their effects on gene expression in the maternal hypothalamus at parturition, folates and 1-carbon metabolites were measured in the brain of the dams. However, the only differences were in brain concentrations of betaine, which were higher in 5X-FA compared to 5X-MTHF dams. These results are consistent with the higher plasma betaine found in 5X-FA compared to 5X-MTHF dams and observations that brain betaine concentrations are mainly reflective of the exchange of betaine between plasma and brain [83]. Brain betaine uptake into neurons only occurs through a betaine transporter that is also responsible for transporting and re-uptake of GABA. As betaine binds with higher affinity to the transporter, higher betaine availability may affect GABA transport/re-uptake and consequential signaling [83,84]. Noteworthy, GABA is the primary inhibitory neurotransmitter in the CNS and GABAergic neurons throughout the hypothalamus have a prominent role in regulating normal food intake, body weight and energy expenditure [85]. It has also been shown that maternal folate diets [86] and common polymorphisms that influence folate status [87] affect GABA availability and signaling in the brain and result in altered gene expression and behavioral patterns. These findings are in line with data derived from our pathway enrichment analysis showing several pathways including GABAergic signaling to be differentially affected in the dam at parturition in response to the folate diet provided during pregnancy. This may suggest a potential role of GABA in the observed folate-mediated effects and warrants future investigation.

A weakness of the study arises from a lack of follow-up on gene expression regulatory mechanisms, including DNA methylation, to explain the observed changes as well as the lack of comparative transcriptomic analyses between recommended vs. high folate gestational diets. Additionally, a time course analysis of metabolic parameters is required to understand critical time-points leading to altered phenotype in the mother and the possible interaction effects of the high fat diet. Furthermore, the results of the present study do not allow a conclusion on whether high MTHF or FA additions to the pregnancy diet are potentially of greater concern. However, a strength of the study is that it raises several important questions regarding the potential adverse programming effects of high MTHF in addition to FA intakes during pregnancy on metabolic programming of mothers. It provides support for more research to understand the pharmacokinetic differences and similarities between the two folate forms throughout various life stages in influencing metabolic disease risk and brain health. More research towards defining an optimal folate dose and form for healthy pregnancy and long-term outcomes should be a priority to inform food regulatory actions pertaining to folate food fortification and prenatal supplements.

## 5. Conclusions

MTHF when compared to FA intake during pregnancy differ in their programing of the early and later-life phenotype of the mother, and a potential adverse role of either form is observed when provided at the higher doses.

## Figures and Tables

**Table 1:** Plasma 5-MTHF and 1-carbon metabolites upon parturition and at 19-weeks post-weaning (PW) of dams fed FA or MTHF pregnancy diets.

	FA						MTHF						Two-Way ANOVA *p*-Value		
	1X-FA			5X-FA			1X-MTHF			5X-MTHF			Dose	Form	Dose × Form
Parturition															
5-MTHF (nmol/L)	103.83	±	18.89	188	±	20.69	117.2	±	18.89	152.6	±	20.69	0.007	0.58	0.24
Methionine (µmol/L)	65	±	4.43	79.2	±	4.85	60.4	±	4.85	76.6	±	4.85	0.005	0.46	0.84
SAM (nmol/L)	297.2	±	19.94	294.3	±	25.74	285.8	±	18.2	281	±	19.94	0.86	0.57	0.96
SAH (nmol/L)	73.2	±	15.09	79.5	±	16.87	123.8	±	15.09	88	±	15.09	0.35	0.07	0.2
Hcy (µmol/L)	14.55	±	3.02	11.9	±	3.31	29.12	±	3.31	20.8	±	3.31	0.11	0.002	0.39
Cys (nmol/L)	1304	±	156.2	1494	±	156.18	1917	±	142.6	1504	±	156.2	0.48	0.06	0.07
Choline (µmol/L)	13.7	±	1.32	12.28	±	1.18	14.42	±	1.08	10.72	±	1.18	0.048	0.73	0.36
Betaine (µmol/L)	34.72 ^ab^	±	2.48	43.12 ^a^	±	2.73	38.72 ^ab^	±	2.49	30.7 ^b^	±	3.04	0.943	0.14	0.007
19-weeks PW															
5-MTHF (nmol/L)	116.9	±	10.29	122.1	±	10.29	131.1	±	11	132.15	±	10.29	0.77	0.26	0.84
Methionine (µmol/L)	76.65	±	4.19	66.9	±	4.84	67.1	±	4.84	74.7	±	4.19	0.81	0.84	0.07
SAM (nmol/L)	255.2	±	22.77	254.6	±	22.77	298.9	±	22.77	279.4	±	22.77	0.66	0.14	0.68
SAH (nmol/L)	72.4	±	8.6	79.77	±	9.19	86.84	±	8.6	75.73	±	8.6	0.82	0.57	0.29
Hcy (µmol/L)	3.69	±	0.98	6.21	±	0.92	4.93	±	0.92	4.51	±	0.92	0.27	0.81	0.13
Cys (nmol/L)	765.47	±	80.91	752.9	±	86.5	733.3	±	80.91	776.36	±	80.91	0.85	0.96	0.74
Choline (µmol/L)	11.23	±	0.97	10.31	±	0.97	11.98	±	0.97	10.44	±	0.97	0.22	0.67	0.75
Betaine (µmol/L)	84.61	±	5.7	78.71	±	5.7	64.44	±	5.7	76.81	±	6.1	0.58	0.07	0.13

Significant at *p* < 0.05 Dose, Form or interaction effect by Two-way repeated measures ANOVA. ^ab^ Significantly different by Tukey Post-hoc Analysis. Values are Least Square Mean ± S.E.M. *n* = 5–6/group at parturition, *n* = 10–12 at 19-weeks post-weaning (PW). Abbreviations: AIN-93G rodent diet with 1X (recommended, 2 mg/kg) or 5X folic acid (FA) or equimolar [6*S*]-5-methyltetrahydrofolic acid calcium salt (MTHF) at 1X (2.1 mg/kg) or 5X (10.4 mg/kg) levels. 5-methyltetrahydrofolate (5-MTHF), Methionine (Met), S-adenosylmethionine (SAM), S-adenosylhomocysteine (SAH), Cysteine (Cys).

**Table 2:** Hepatic 5-MTHF and 1-carbon metabolites upon parturition and at 19-weeks post-weaning (PW) of dams fed FA or MTHF pregnancy diets.

	FA						MTHF						Two-Way ANOVA *p*-Value		
	1X-FA			5X-FA			1X-MTHF			5X-MTHF			Dose	Form	Dose × Form
Parturition															
5-MTHF (nmol/g)	26.13	±	2.92	25.67	±	2.92	23.73	±	2.92	26.69	±	2.92	0.68	0.81	0.56
Met (nmol/g)	181.5	±	14.65	212.67	±	14.66	181.2	±	16.05	220.25	±	17.95	0.04	0.82	0.81
SAM (nmol/g)	55.5	±	4.27	51.55	±	3.48	54.08	±	3.82	51.77	±	4.27	0.44	0.88	0.83
SAH (nmol/g)	31.76	±	1.56	30.68	±	1.56	34.7	±	1.71	35.52	±	1.92	0.94	0.03	0.58
Cys (nmol/g)	20.2 ^ab^	±	3.81	18.82 ^ab^	±	3.81	13.25 ^a^	±	4.26	31.42 ^b^	±	4.26	0.06	0.49	0.03
Choline (nmol/g)	1759 ^a^	±	154.86	1124.17 ^b^	±	141.37	787.5 ^b^	±	154.86	988.75 ^b^	±	173.14	0.2	0.004	0.02
Betaine (nmol/g)	1078	±	163.18	1247.5	±	148.96	1156	±	163.18	793.73	±	182.4	0.57	0.27	0.13
19-weeks PW															
5-MTHF (nmol/g)	26.13	±	2.92	25.67	±	2.92	23.73	±	2.92	26.69	±	2.92	0.68	0.81	0.56
Met (nmol/g)	424.75	±	50.01	419.63	±	50.01	475	±	50.01	489.75	±	50.01	0.92	0.24	0.84
SAM (nmol/g)	19.21 ^ab^	±	3.02	28.07 ^a^	±	2.61	26.96 ^ab^	±	2.79	18.1 ^b^	±	2.62	0.99	0.69	0.004
SAH (nmol/g)	25.98	±	2.87	33.5	±	2.48	36.07	±	2.65	28.92	±	2.48	0.99	0.30	0.01
Cys (nmol/g)	21.4	±	1.59	19.65	±	1.59	20.07	±	1.7	20.21	±	1.59	0.62	0.81	0.56
Choline (nmol/g)	557.75	±	66.24	635.25	±	66.62	746.12	±	66.62	626.25	±	66.62	0.75	0.19	0.14
Betaine (nmol/g)	2938.75	±	338.44	2552.5	±	338.44	2185.71	±	361.81	2440.88	±	338.44	0.85	0.22	0.36

Significant at *p* < 0.05 Dose, Form or Dose×Form interaction effect by Two-way repeated measures ANOVA. ^ab^ Significantly different by Tukey Post-hoc Analysis. Values are Least Square Mean ± S.E.M. *n* = 16–18/group up parturition, *n* = 10–12 up to 19-weeks post-weaning (PW). Abbreviations: AIN-93G rodent diet with 1X (recommended, 2 mg/kg) or 5X folic acid (FA) or equimolar [6*S*]-5-methyltetrahydrofolic acid calcium salt (MTHF) at 1X (2.1 mg/kg) or 5X (10.4 mg/kg) levels. 5-MTHF, 5-methyltetrahydrofolate; Met, methionine; SAM, S-adenosylmethionine; SAH, S-adenosylhomocysteine; Cys, Cysteine.3.2. Effects of Folate Diets on Litter Size, Body Composition and Food Intake.

**Table 3:** Body composition upon parturition and at 19-weeks post-weaning (PW) of dams fed FA or MTHF pregnancy diets.

	FA						MTHF						Two-Way ANOVA *p*-Value		
	1X-FA			5X-FA			1X-MTHF			5X-MTHF			Dose	Form	Dose × Form
Parturition															
AI (%)	2.84	±	0.33	2.81	±	0.5	3.63	±	0.68	2.75	±	0.41	0.39	0.48	0.41
Liver (%)	4.48 ^ab^	±	0.14	4.18 ^ab^	±	0.11	4.09 ^a^	±	0.08	4.66 ^b^	±	0.17	0.35	0.76	0.006
19-weeks PW															
AI (%)	9.6	±	0.7	8.9	±	0.7	9.9	±	0.6	9	±	0.5	0.19	0.85	0.83
Liver (%)	2.41	±	0.12	2.21	±	0.09	2.33	±	0.09	2.29	±	0.11	0.23	0.81	0.33
Total liver lipids (%)	5.32	±	0.26	5.12	±	0.25	5.7	±	0.25	5.25	±	0.25	0.21	0.32	0.63

Significant at *p* < 0.05 Dose, Form or Dose×Form interaction effect by Two-way repeated measures ANOVA. ^ab^ Significantly different by Tukey Post-hoc Analysis. Values are Least Square Mean ± S.E.M. *n* = 16–18/group up parturition, *n* = 10–12 up to 1-week PW. Abbreviations: AIN-93G rodent diet with 1X (recommended, 2 mg/kg) or 5X folic acid (FA) or equimolar [6*S*]-5-methyltetrahydrofolic acid calcium salt (MTHF) at 1X (2.1 mg/kg) or 5X (10.4 mg/kg) levels. AI, Adiposity Index: percentage of total visceral adipose tissue weight adjusted per gram of body weight.

**Table 4:** Plasma glucose and hormones upon parturition and at 19-weeks post-weaning (PW) of dams fed FA or MTHF pregnancy diets.

	FA						MTHF						Two-Way ANOVA *p*-Value		
	1X-FA			5X-FA			1X-MTHF			5X-MTHF			Dose	Form	Dose × Form
Parturition															
Leptin ng/mL	5.68	±	0.74	6.13	±	0.74	4.39	±	0.81	3.88	±	0.81	0.97	0.03	0.55
Leptin/VAT	0.63	±	0.12	0.88	±	0.12	0.43	±	0.13	0.5	±	0.13	0.23	0.03	0.46
Insulin ng/mL	1.36	±	0.34	1.88	±	0.31	1.14	±	0.31	1.46	±	0.31	0.20	0.33	0.77
Glucose mg/dL	116.8	±	6.54	122.4	±	5.85	101	±	5.85	115.1	±	5.85	0.12	0.08	0.50
HOMA-IR	1.67	±	0.37	2.31	±	0.33	1.19	±	0.33	1.71	±	0.33	0.11	0.13	0.86
19-weeks PW															
Leptin ng/mL	9.9	±	2.06	9.66	±	1.71	15.02	±	1.71	14.96	±	1.86	0.94	0.008	0.96
Leptin/VAT	0.23	±	0.06	0.26	±	0.05	0.39	±	0.06	0.44	±	0.06	0.53	0.007	0.88
Ghrelin ng/mL	146.6	±	16	120.1	±	14.3	78.73	±	16	96.96	±	15.1	0.79	0.006	0.16
Insulin ng/mL	1.75	±	0.22	1.62	±	0.23	1.98	±	0.23	1.71	±	0.26	0.40	0.52	0.77
Glucose mg/dL	129.9	±	2.39	128.5	±	2.53	128.2	±	2.71	123.9	±	2.71	0.27	0.21	0.57
HOMA-IR	2.56	±	0.3	2.35	±	0.32	2.64	±	0.3	2.2	±	0.35	0.30	0.89	0.76

Significant at *p* < 0.05 Dose, Form or Dose×Form interaction effect by Two-way repeated measures ANOVA. Values are Least Square Mean ± S.E.M. *n* = 5–6/group at parturition, *n* = 10–12 at 19-weeks PW. Abbreviations: AIN-93G rodent diet with 1X (recommended, 2 mg/kg) or 5X folic acid (FA) or equimolar [6*S*]-5-methyltetrahydrofolic acid calcium salt (MTHF) at 1X (2.1 mg/kg) or 5X (10.4 mg/kg) levels. VAT, visceral adipose tissue; HOMA-IR, homeostatic model assessment insulin resistance calculated as follows: [fasting glucose (in mg/dL) × fasting insulin (in µU/mL)]/2430].

**Table 5:** Brain 5-MTHF and 1-carbon metabolites upon parturition and at 19-weeks post-weaning (PW) of dams fed 5X-FA or 5X-MTHF pregnancy diets.

	5X-FA			5X-MTHF			*p*-Value
Parturition							
5-MTHF (nmol/g)	0.24	±	0.02	0.29	±	0.05	0.43
Met (nmol/g)	48.9	±	1.21	44.66	±	2.07	0.14
SAM (nmol/g)	22.57	±	0.5	21.86	±	1.06	0.59
SAH (nmol/g)	3.4	±	0.34	3	±	0.19	0.34
Cys (nmol/g)	47.95	±	4.87	46.12	±	5.84	0.82
Choline (nmol/g)	811	±	51.9	706	±	38.02	0.14
Betaine (nmol/g)	13.65	±	2.25	6.36 *	±	1.15	0.02
19-weeks PW							
5-MTHF (nmol/g)	0.26	±	0.03	0.23	±	0.03	0.59
Met (nmol/g)	41.4	±	3.66	36.5	±	4.61	0.43
SAM (nmol/g)	21.07	±	0.81	22.1	±	1.63	0.86
SAH (nmol/g)	2.77	±	0.18	2.58	±	0.12	0.42
Cys (nmol/g)	42.2	±	1.92	44.46	±	2.82	0.53
Choline (nmol/g)	408.83	±	40.72	375	±	22.27	0.14
Betaine (nmol/g)	8.94	±	1.08	7.16	±	0.46	0.51

* Significant at *p* < 0.05 by *t*-test. Values are Mean ± S.E.M. *n* = 5–6/group at parturition, *n* = 6–7 at 19-weeks PW. Abbreviations: AIN-93G rodent diet with 5X folic acid (FA) or equimolar [6*S*]-5-methyltetrahydrofolic acid calcium salt (MTHF, 10.4 mg/kg) levels. 5-MTHF, 5-methyltetrahydrofolate; Met, methionine; SAM, S-adenosylmethionine; SAH, S-adenosylhomocysteine; Cys, Cysteine.

## Data Availability

The RNAseq data presented in this study are openly available in NCBI Gene Expression Omnibus (GEO) at [https://www.ncbi.nlm.nih.gov/geo/query/acc.cgi?acc=GSE161954], reference number [GSE161954].

## Supplementary Materials

The following are available online at https://www.mdpi.com/2072-6643/13/1/48/s1, Figure S1: Weekly weight-gain of dams from weaning to 19-weeks post-weaning (PW). Figure S2: Insulin Resistance at 1-week post-weaning in dams fed FA or MTHF pregnancy diets. Figure S3: Locomotor activity at 15-weeks post-weaning in dams fed FA or MTHF pregnancy diets. Figure S4: Comparison between data derived from RNAseq and qRT-PCR in ARC at parturition of dams fed 5X-FA or 5X-MTHF pregnancy diets. Table S1: Composition of experimental folate diets fed during pregnancy to Wistar rat dams and post-weaning high fat diet (HFD). Table S2: List of primers used for gene expression assay in hypothalamus of 5X-FA vs. 5X-MTHF dams at parturition and post-weaning. Table S3: Body weight and food intake changes up to 1-week (wk) post-weaning (PW) of dams fed FA or MTHF diets during pregnancy. Table S4: Differentially Expressed Genes (DEGs) derived from DeSeq. Up- and Down-regulated gene list with *padj* < 0.05 were used for pathway enrichment. Table S5: Up-regulated, down-regulated or up-and down-regulated gene lists classified by GO enrichment Biological Processes (BP), Cellular Component (CC), and Molecular Function (MF). Table S6: Up-regulated, down-regulated or up-and down-regulated gene lists classified by GO Biological Processes (BP) using Markov Cluster (MCL) algorithm. Table S7: Up-regulated, down-regulated or up-and down-regulated gene lists classified by KEGG and Reactome pathways. Table S8: Common genes between our differentially expressed gene lists (*padj* < 0.05) and those identified in the disease-centre human database, Human Genome Epidemiology (HuGE) Navigator.
