# Supplementary material for: [6S]-5-Methyltetrahydrofolic Acid and Folic Acid Pregnancy Diets Differentially Program Metabolic Phenotype and Hypothalamic Gene Expression of Wistar Rat Dams Post-Birth
Source: Nutrients. 2020 Dec 25;13(1):48. doi: 10.3390/nu13010048 (PMC7823556; doi:10.3390/nu13010048)
Supplement: Supplementary file 1 [file nutrients-13-00048-s001.zip › FigureS1 to S4_Pannia et al 2020_Dec 11.pptx]

## Slide 1
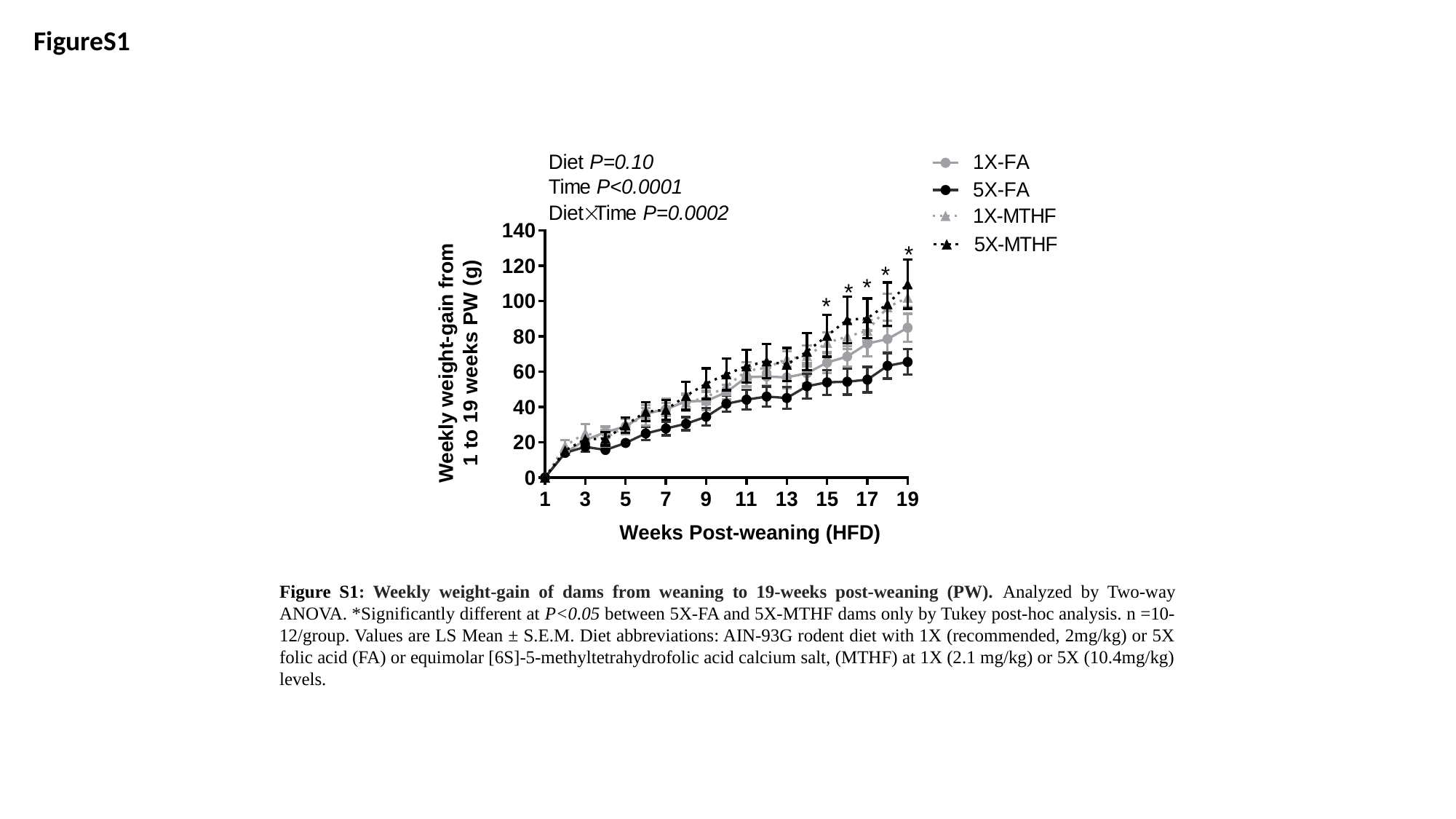

FigureS1
Figure S1: Weekly weight-gain of dams from weaning to 19-weeks post-weaning (PW). Analyzed by Two-way ANOVA. *Significantly different at P<0.05 between 5X-FA and 5X-MTHF dams only by Tukey post-hoc analysis. n =10-12/group. Values are LS Mean ± S.E.M. Diet abbreviations: AIN-93G rodent diet with 1X (recommended, 2mg/kg) or 5X folic acid (FA) or equimolar [6S]‐5‐methyltetrahydrofolic acid calcium salt, (MTHF) at 1X (2.1 mg/kg) or 5X (10.4mg/kg) levels.

## Slide 2
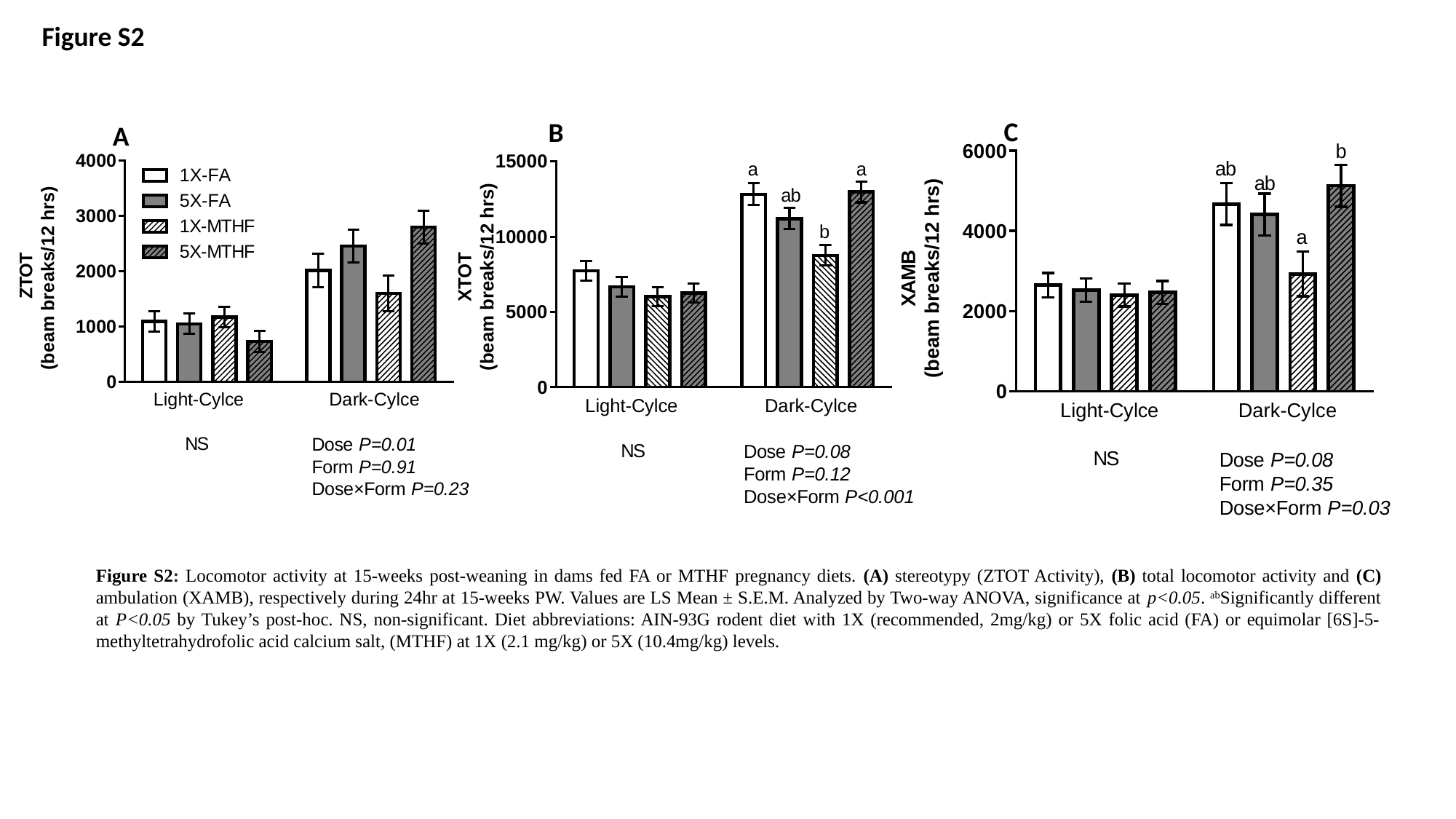

Figure S2
C
B
A
Figure S2: Locomotor activity at 15-weeks post-weaning in dams fed FA or MTHF pregnancy diets. (A) stereotypy (ZTOT Activity), (B) total locomotor activity and (C) ambulation (XAMB), respectively during 24hr at 15-weeks PW. Values are LS Mean ± S.E.M. Analyzed by Two-way ANOVA, significance at p<0.05. abSignificantly different at P<0.05 by Tukey’s post-hoc. NS, non-significant. Diet abbreviations: AIN-93G rodent diet with 1X (recommended, 2mg/kg) or 5X folic acid (FA) or equimolar [6S]‐5‐methyltetrahydrofolic acid calcium salt, (MTHF) at 1X (2.1 mg/kg) or 5X (10.4mg/kg) levels.

## Slide 3
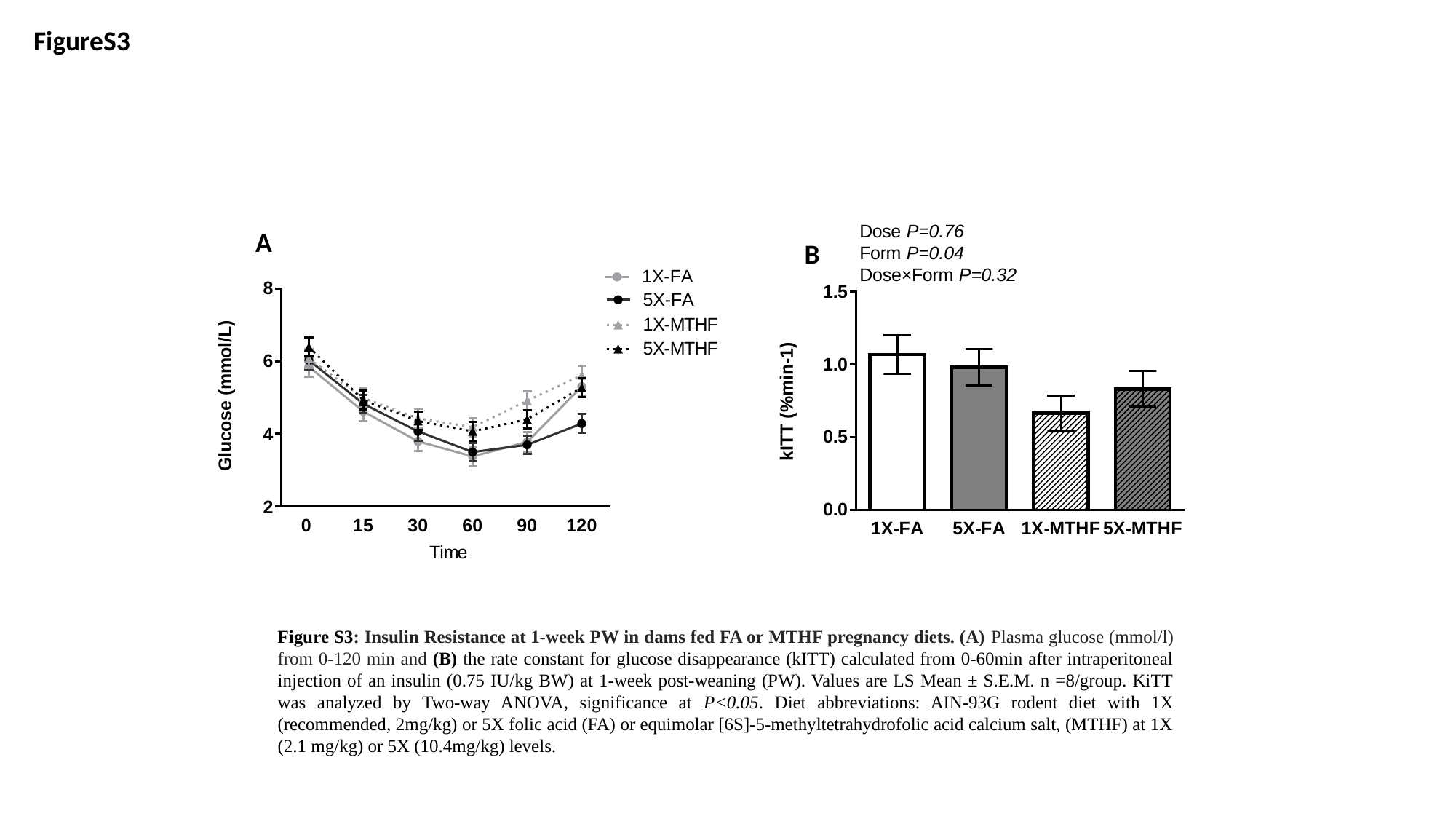

FigureS3
A
B
Figure S3: Insulin Resistance at 1-week PW in dams fed FA or MTHF pregnancy diets. (A) Plasma glucose (mmol/l) from 0-120 min and (B) the rate constant for glucose disappearance (kITT) calculated from 0-60min after intraperitoneal injection of an insulin (0.75 IU/kg BW) at 1-week post-weaning (PW). Values are LS Mean ± S.E.M. n =8/group. KiTT was analyzed by Two-way ANOVA, significance at P<0.05. Diet abbreviations: AIN-93G rodent diet with 1X (recommended, 2mg/kg) or 5X folic acid (FA) or equimolar [6S]‐5‐methyltetrahydrofolic acid calcium salt, (MTHF) at 1X (2.1 mg/kg) or 5X (10.4mg/kg) levels.

## Slide 4
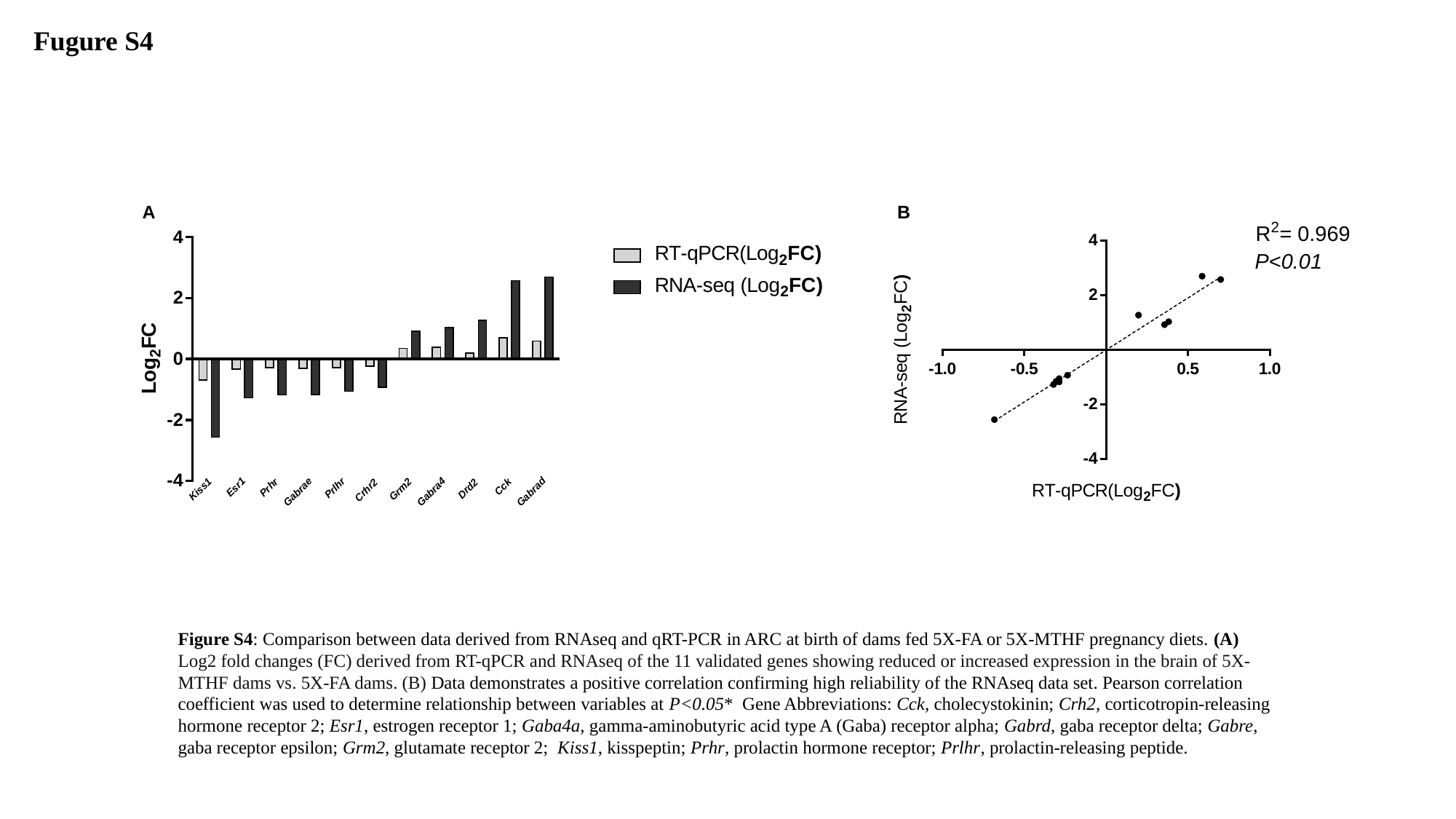

Fugure S4
B
A
Figure S4: Comparison between data derived from RNAseq and qRT-PCR in ARC at birth of dams fed 5X-FA or 5X-MTHF pregnancy diets. (A) Log2 fold changes (FC) derived from RT-qPCR and RNAseq of the 11 validated genes showing reduced or increased expression in the brain of 5X-MTHF dams vs. 5X-FA dams. (B) Data demonstrates a positive correlation confirming high reliability of the RNAseq data set. Pearson correlation coefficient was used to determine relationship between variables at P<0.05* Gene Abbreviations: Cck, cholecystokinin; Crh2, corticotropin-releasing hormone receptor 2; Esr1, estrogen receptor 1; Gaba4a, gamma-aminobutyric acid type A (Gaba) receptor alpha; Gabrd, gaba receptor delta; Gabre, gaba receptor epsilon; Grm2, glutamate receptor 2; Kiss1, kisspeptin; Prhr, prolactin hormone receptor; Prlhr, prolactin-releasing peptide.
